# Multi-drug resistance and extended spectrum beta lactamase producing Gram negative bacteria from chicken meat in Bharatpur Metropolitan, Nepal

**DOI:** 10.1186/s13104-017-2917-x

**Published:** 2017-11-07

**Authors:** Anil Shrestha, Anup Muni Bajracharya, Hemraj Subedi, Raju Shah Turha, Sachin Kafle, Saroj Sharma, Sunil Neupane, Dhiraj Kumar Chaudhary

**Affiliations:** 1 Department of Microbiology, Balkumari College, Chitwan, Nepal; 2 Department of Microbiology, Prithu Technical College, Institute of Agriculture and Animal Science, Tribhuvan University, Dang, Nepal

**Keywords:** Gram negative bacteria, Extended spectrum beta lactamase, Multidrug resistance, Chicken meat, Antibiotics

## Abstract

**Objective:**

Multidrug resistance (MDR) and extended spectrum beta lactamase (ESBL) producer Gram negative bacteria are considered as a major health problem, globally. ESBL enzyme hydrolyses the beta lactam ring of third generation cephalosporins, which alters the structure of the antibiotic. Due to the modification in structure of the antibiotic, bacteria show resistance to these antibiotics. Resistant bacterial strains are transmitted to humans from animals through consumption of uncooked meat, through contact with uncooked meat and meat surfaces. This study aims to assess bacteriological profile and analyze the situation of antibiotic resistance, multidrug resistance, and ESBL producing Gram negative bacteria in chicken meat.

**Results:**

A total of 38 chicken meat samples were studied in which 103 Gram negative bacteria were isolated. Species of Gram negative bacteria were identified as *Citrobacter* spp. (44.7%), *Salmonella* spp. (26.2%), *Proteus* spp. (18.4%), *Escherichia coli* (4.8%), *Shigella* spp. (3.9%), *Pseudomonas* spp. (1.9%), and *Klebsiella* spp. (1.0%). The prevalence of MDR isolates was found to be 79.6%. Total ESBL producer was 36.9% and ESBL producer among MDR was 34.9%. This concludes wide range of antibiotic resistance bacteria is prevalent in raw chicken meat.

**Electronic supplementary material:**

The online version of this article (10.1186/s13104-017-2917-x) contains supplementary material, which is available to authorized users.

## Introduction

Multidrug resistance (MDR) is the ability of bacteria to resist different classes of antibiotics (three or more than three classes of antibiotics) which are structurally different and have different molecular targets [1]. Antibiotic resistance is a result of antibiotic use. The greater the volume of antibiotics used, the greater will be the chances of arising antibiotic resistance population of bacteria [2]. There is growing evidence which revealed antibiotic resistance has been promoted by widespread use of non-therapeutic antibiotics in animals [3]. The misuse of antibiotic can lead to the development of bacterial resistance towards antibiotic, increases the burden of chronic disease, and increases costs of health services. Resistant bacteria are transmitted to human through direct contact with animal, by exposure to animal manure, through consumption of uncooked meat, and through contact with meat surfaces [4].

The prevalence of MDR isolates and ESBL producing isolates is increasing in humans as well as animal. Fecal carriage of ESBL gene has been identified as the major reservoir in the environment. Bacterial species that carry ESBL genes are normal inhabitants of gastrointestinal tract, and food is a potential source of them [5]. Meat harbor different bacteria as an inherent contamination and are further contaminated during handling, improper dressing, cleaning, insanitary condition, and unhygienic practices of selling meat. Consumption of these unsafe meat arise public health hazards [6, 7]. This study aims to find the prevalence of MDR and ESBL producing isolates from chicken meat in Bharatpur metropolitan.

## Main text

This cross-sectional study was conducted in Microbiology Laboratory of Balkumari College, Tribhuvan University, Bharatpur, Nepal from 2016 December to 2017 June. Random sampling was done to collect non-repeated single meat sample from different slaughter house located in different places of Bharatpur, Nepal. Sample size was determined based on prevalence rate as reported by previous study [8]. A total of 38 samples of chicken meat were included in this study.

## Methodology

The chicken meat samples (15 g; each 5 g from thighs, breasts, and wings of same chicken) prior to refrigeration were aseptically collected in a sterile beaker. The beaker was properly capped with aluminum foil and transported quickly to the laboratory. Samples were transferred to conical flask containing peptone water and incubated for 30 min at 80 rpm at room temperature in a rotator (Thermo Scientific Compact Digital Mini Rotator; Cat. No. 8880025). After incubation, 1 ml of contaminated peptone water (HiMedia, M028) was further transferred to two different test tubes containing nutrient broth (HiMedia, MM244) and Selenite F broth (Himedia, M025S). Test tubes were incubated aerobically at 37 °C overnight. After incubation, samples from nutrient broth were streaked in m-endo agar (HiMedia, M1106) and MacConkey agar plates (HiMedia, M081). Samples from Selenite F broth were streaked to Salmonella-Shigella agar plates (HiMedia, M108). All the plates were incubated aerobically at 37 °C for 24 h. Gram negative isolates were identified by following standard microbiological techniques which include studies of colony morphology, staining reactions and various biochemical properties [9]. Pure isolates were identified by performing the standard biochemical tests (SIM test, MRVP test, citrate test, urease test) [8].

Antibiotic susceptibility test of isolates was performed following modified Kirby-bauer disk diffusion method as recommended by Clinical and Laboratory Standards Institute (CLSI) [10]. The antibiotics used in this study were nitrofurantoin (NIT), ampicillin (Amp), cefotaxime (CTX), ceftazidime (CAZ), gentamicin (Gen), ciprofloxacin (Cip), colisitin (Cl), doxycycline hydrochloride (DO), imipenam (Imp) and polymyxin B (Pb). Screening of ESBL was performed by using ceftazidime (30 µg) and cefotaxime (30 µg) disks. The zone of inhibition (ZOI) ≤ 22 mm for ceftazidime and 27 mm for cefotaxime was considered as potential ESBL producer as recommended by CLSI. For the conformation of ESBL, combination disk method was used. The combination of ceftazidime and cefotaxime alone and in combination with clavulanic acid (CA) (10 µg) were used. An increase ZOI of ≥ 5 mm for either antimicrobial agent tested in combination with CA versus its zone when tested alone confirms ESBL positive. All the antibiotic discs used in this study were purchased from Himedia, India. For biochemical tests and antibiotics susceptibility tests, following reference strains were used as quality control strains: *E coli* ATCC 25922; *Pseudomonas aeruginosa* ATCC 27853; *Klebsiella pneumonia* ATCC 700603; *Proteus mirabilis* ATCC 35659; and *Salmonella typhimurium* ATCC 14028.

All data were entered in Microsoft Excel and *Chi* square test was performed. *P* value was calculated and considered significant only when it was less than 0.05.

## Results

**Fig. 1:**
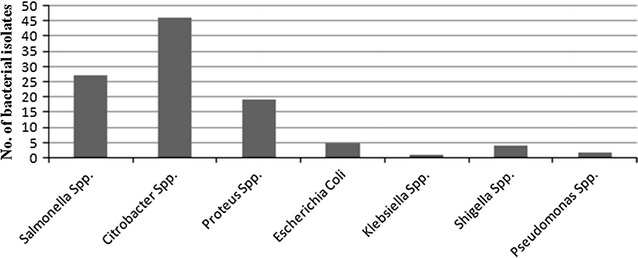
Distribution of bacterial isolates in chicken meat samples

Based on sanitation parameters (hygienic condition of slaughter house, chopping boards, and showcased meat, and cleanliness of slaughter personnel), we have categorized meat samples as good sanitation and poor sanitation type. Out of 38 collected samples, 14 samples were grouped into good sanitation type and 24 samples were grouped into poor sanitation type (Additional file 1: Table S1). A total of 103 Gram negative bacterial strains were isolated among which only one sample showed growth with single isolate and remaining all samples showed growth with multiple isolates. Out of 103 bacterial isolates, 27 (26.2%) and 76 (73.8%) bacterial isolates were obtained from good sanitation and poor sanitation type meat samples, respectively (Additional file 1: Table S2). Among 103 Gram negative isolates, the number of *Citrobacter* spp., *Salmonella* spp., *Proteus* spp., *Escherichia coli*, *Klebsiella* spp., *Shigella* spp. and *Pseudomonas* spp. were 46 (44.7%), 26 (26.2%), 19 (18.4%), 5 (4.8%), 1 (1.0%), 4 (3.9%) and 2 (1.9%), respectively (Fig. 1).

Among 103 Gram negative isolates, 36 (34.9%) of bacteria were MDR producing extended spectrum beta lactamase, in which 13 (12.6%) strains of *Salmonella* spp. account to be highest MDR ESBL producer (Table 1). From both good sanitation and poor sanitation type meat samples, 82 (79.6%) isolates were detected as multidrug resistance. Among MDR isolates, *Salmonella* spp. (85.2%) and *Pseudomonas* spp. (100.0%) showed high prevalent of MDR. The frequency of ESBL producer bacteria in meat samples was found to be 38 (36.9%). *Pseudomonas* spp. (100.0%), *E. coli* spp. (40.0%), and *Salmonella* spp. (55.2%) showed high prevalent of ESBL producer bacteria (Table 2). Statistical analysis showed that there was no significant association in prevalence of MDR and ESBL producer isolates with sanitation condition of meat samples (*P* > 0.05).

**Table 1:** Frequency of multi-drug resistance ESBL producing bacteria

Bacteria	Multidrug resistance bacteria producing ESBL (%)
*Citrobacter* spp.	11 (10.7)
*Salmonella* spp.	13 (12.6)
*Proteus* spp.	5 (4.9)
*E. coli*	2 (1.9)
*Shigella* spp.	3 (2.9)
*Pseudomonas* spp.	2 (1.9)
Total	36 (34.9)

**Table 2:** Frequency of MDR and ESBL producing bacteria

Bacteria	Multi-drug resistance bacteria (%)	ESBL producer bacteria (%)
*Citrobacter* spp. (n = 46)	36 (78.3)	12 (26.1)
*Salmonella* spp. (n = 27)	23 (85.2)	15 (55.2)
*Proteus* spp. (n = 19)	14 (73.7)	5 (26.3)
*E. coli* (n = 5)	4 (80.0)	2 (40.0)
*Shigella* spp. (n = 4)	3 (75.0)	2 (40.0)
*Pseudomonas* spp. (n = 2)	2 (100.0)	2 (100.0)
Total	82 (79.6)	38 (36.9)

The antibiotic resistance pattern of the *Citrobacter* spp. showed highest 32.6% isolates were resistant to the imipenam followed by cefotaxime and ciprofloxacin (19.5%, each). All the strains of *Salmonella* spp. were resistant to ampicillin (100%) followed by nitofurantoin (84.6%) and doxycycline hydrochloride (84.0%). *Proteus* spp. revealed 29.4% resistivity to imipenam followed by ciprofloxacin (11.7%) (Additional file 1: Table S3). The antibiotic resistivity pattern of *E. coli* showed all the isolates were resistant to ampicilin followed by colistin and polymyxin B (80%, each), whereas resistivity were not detected to doxycycline hydrochloride, gentamicin and imipenam.

## Discussion

Animals and its products are potent source of MDR bacteria. Consumption of unhealthy meat, unhygienic livestock practices and polluted environment surrounding slaughter house contribute for transmission of several diseases and antibiotic resistant bacterial strains [4, 11]. In Nepal, information regarding prevalence of MDR and ESBL producer Gram negative bacteria in chicken meat is poorly available. Therefore, this study was conducted to assess prevalence of MDR and ESBL producer Gram negative bacteria in chicken meat.

This study observed that *Citrobacter* spp., *Salmonella* spp., *Proteus* spp., *E. coli*, *Klebsiella* spp., *Shigella* spp. and *Pseudomonas* spp. were the major Gram negative bacteria among 103 bacterial isolates (Fig. 1). Most of these isolates are considered as pathogenic which suggest chicken meat is an important source of food borne infection. Similar pattern of Gram negative bacteria were found in several other studies conducted in North East India, China, South Korea, Vietnam, and Spain [11–15].

*Citrobacter* species are frequently found in water, soil, food, and the intestines of animals and humans. Most human cases of *Citrobacter* infection are caused by *Citrobacter freundii* and *Citrobacter koseri* [16, 17]. The prevalence of MDR and ESBL producing *Citrobacter* spp. was found to be 78.3% and 26.1%, respectively. These magnitudes are comparable with the study conducted by Kanamori et al. which reported 19.3% of *Citrobacter* spp. was ESBL producer [18]. *Citrobacter* spp. is a low virulence bacterium and thus can persist in host population for long periods. Over time, they accumulate resistance determinants which may transform to more virulent organisms [17].

The prevalence of *Salmonella* spp. was 26.2% which is similar to the studies carried out in USA and South Korea [13, 19]. In contrast, higher prevalence rate of *Salmonella* spp. was found in Southern Thailand (67.5%) [20] and China (54.0%) [12]. Prevalence of *Salmonella* spp. in chicken meat indicates that contamination may occur during slaughtering process or evisceration. *Salmonella* spp. in chicken meat can be considered as important cause of food borne Salmonellosis [13, 21]. *Salmonella* spp. isolates from this study were resistant to ampicillin (100.0%), nitrofurantion (84.6%) and doxycycline hydrochloride (84.0%). Similar broad resistant pattern were observed in previous studies [22, 23]. This study showed the prevalence of MDR and ESBL producing *Salmonella* spp. was 85.2% and 55.2%, respectively. In a study conducted in South Korea found 87.2% of *Salmonella* spp. were MDR isolates [13]. In Thailand, 84.4% isolates of *Salmonella* spp. were multidrug resistant which were isolated from chicken meat [19]. The prevalence of ESBL producing *Salmonella* spp. was very high compared to study performed by Wu et al. [24], which reported only 8.6% prevalence of ESBL producing *Salmonella* species. Attention should be given to control the presence of high rate of ESBL-producing *Salmonella* in food.

Prevalence of *E. coli* MDR and ESBL producer strains were 80.0% and 40%, respectively. Similar resistivity pattern was observed in Vietnam [14], China [25], Portugal [26], and Spain [15]. In addition, several studies have reported an increased ESBL cases from *E. coli* strains isolated from animals and pets [15, 26, 27]. *Escherichia coli* are common inhabitants of the human and animal guts and are indicators of fecal contamination in food. However, they have also emerged as important causes of nosocomial and community-acquired infections [28].

The MDR pattern of *Proteus* spp. showed 14 (73.7%) isolates were multidrug resistance. Among all *Proteus* spp., 5 (26.3%) isolates were ESBL producer. Numerous studies have reported presence of MDR strains *Proteus* species from animal sources [29, 30].

In overall, we found high prevalence (79.6%) of MDR bacteria in chicken meat. It is well documented that Gram negative bacilli harbor series of antibiotic resistant genes which can be transferred to other bacteria horizontally [31]. All Gram negative bacilli isolated in this study namely *E. coli, Salmonella* spp. have been shown to cause different nosocomial infection [32]. This indicates that the emergence of MDR strains from chicken meat is potent threat. In this study, the prevalence of ESBL producer bacteria in chicken meat is 36.9% and ESBL producer among MDR was 34.9%. ESBL production by the bacteria might be higher due to excessive use of broad spectrum antibiotics. The ESBL enzymes are mutant, plasmid-mediated beta lactamases derived from older, broad spectrum beta lactamase (e.g. TEM-1, TEM-2, SHV-1). Thus, they mediate resistance to extended spectrum (third generation) Cephalosporins (e.g., Ceftazidime, Cefotaxime, Ceftriaxone) [33].

## Conclusions

This study showed high prevalence of MDR and ESBL producer Gram negative bacterial strains in chicken meat. The frequency of Gram negative bacteria as MDR and ESBL producer is elevating globally. Antibiotic resistance is a worldwide problem and its transmission from animal source to human is increasing tremendously. Both MDR and ESBL incidence are considered as extreme public health issue. The problem of the bacterial resistance to antimicrobial drugs is more troublesome to developing countries like Nepal where facilities for health care, surveillance for antibiotics medication and facilities to detect MDR and ESBL are poorly developed.

## Limitations

Gram positive bacteria, yeast, and fungi were not considered in this study. We were unable to assess the quality of water used for washing purpose, and sanitation condition of the site where birds were undressed and eviscerated. Future studies should consider these factors in addressing the MDR and ESBL prevalence.

## Additional file

**Additional file 1.** Additional tables and figures.

## Abbreviations

MDR: multidrug resistance
ESBL: extended spectrum beta lactamase
SIM: sulfide, indole, motility
MRVP: methyl-red, Voges Proskauer
CLSI: Clinical and Laboratory Standards Institute
ZOI: zone of inhibition
