# Supplementary material for: Multi-drug resistance and extended spectrum beta lactamase producing Gram negative bacteria from chicken meat in Bharatpur Metropolitan, Nepal
Source: BMC Res Notes. 2017 Nov 7;10:574. doi: 10.1186/s13104-017-2917-x (PMC5678746; doi:10.1186/s13104-017-2917-x)
Supplement: Supplementary file 1 — Additional file 1. Additional tables and figures. [file 13104_2017_2917_MOESM1_ESM.pdf]

Additional file 1

BMC Research Notes

Multi-drug resistance and extended spectrum beta lactamase producing Gram negative bacteria from chicken meat in Bharatpur Metropolitan, Nepal

Anil Shrestha[1], Anup Muni Bajracharya[1], Hemraj Subedi[1], Raju Shah Turha[1], Sachin Kafle[1], Saroj Sharma[1], Sunil Neupane[1] and Dhiraj Kumar Chaudhary[2]*

[1]Department of Microbiology, Balkumari College, Chitwan, Nepal

[2]Department of Microbiology, Prithu Technical College, Institute of Agriculture and Animal Science, Tribhuvan University, Dang, Nepal

Contents category: Research note

***Corresponding author:** Dhiraj Kumar Chaudhary

Tel: +977-9841441236

E-mail: dhirajchaudhary2042@gmail.com

Additional Table S1: Questionnaire during sample collection. R, regular; Ir, irregular; C, covered; UC, uncovered; F, flies observed.

Sample	Washing of slaughter house		Washing of apron		Use of Handsanitizer	Washing chopping board		Showcased meat condition			Sanitation type
	R	Ir	R	Ir		R	I	C	UC	F	
B-1	√		√		No	√		√		No	Good
B-2	√		√		Yes	√		√		No	Good
B-3		√		√	No		√		√	Yes	Poor
B-4		√		√	No		√		√	Yes	Poor
B-5	√			√	No	√		√		No	Good
B-6		√		√	No		√		√	Yes	Poor
B-7	√			√	Yes	√		√		No	Good
B-8		√		√	No		√		√	Yes	Poor
B-9		√		√	No		√		√	Yes	Poor
B-10		√	√		No	√		√		No	Good
B-11		√		√	No	√			√	Yes	Poor
B-12	√			√	No	√		√		No	Good
B-13		√		√	No		√		√	Yes	Poor
B-14		√		√	No		√		√	Yes	Poor
B-15		√		√	No		√		√	Yes	Poor
B-16		√		√	No	√			√	Yes	Poor
B-17		√		√	No		√		√	Yes	Poor
B-18		√	√		No	√		√		No	Good
B-19		√		√	No		√		√	Yes	Poor
B-20		√		√	No		√		√	Yes	Poor
B-21	√		√		No	√		√		No	Good
B-22		√		√	No	√			√	Yes	Poor
B-23	√		√		Yes	√		√		No	Good

B-24		√		√	No		√		√	Yes	Poor
B-25		√		√	No		√		√	Yes	Poor
B-26		√		√	No		√		√	Yes	Poor
B-27	√		√		No	√		√		No	Good
B-28		√		√	No		√		√	Yes	Poor
B-29	√			√	No	√		√		No	Good
B-30		√		√	No		√		√	Yes	Poor
B-31		√		√	No	√			√	Yes	Poor
B-32		√		√	No	√			√	Yes	Poor
B-33		√		√	No		√		√	Yes	Poor
B-34	√			√	No	√		√		No	Good
B-35		√		√			√		√		Poor
B-36	√		√		Yes	√		√		No	Good
B-27		√	√		No	√		√		No	Good
B-38		√		√			√		√		Poor

Additional Table S2: Number of bacterial isolates in different chicken meat samples.

Sample type	No. of bacterial isolates per sample	No. of sample	Total no. of bacterial isolates
Good Sanitation	1	1	1
	2	13	26
Poor sanitation	3	20	60
	4	4	16
Total		38	103

Additional Table S3: Antibiotic resistivity pattern of major isolated bacteria.

Antibiotic Group	Antibiotic Used	*Citrobacter* species (%)	*Salmonella* species (%)	*Proteus* species (%)
Cephalosporin	Cefotaxime	19.5	5.9	5.8
	Ceftazidime	18.7	12.5	0
Aminoglycoside	Gentamicin	5.1	2.5	0
Carbapenems	Imipenem	32.6	33.3	29.4
Fluoroquinolones	Ciprofloxacin	19.5	46.1	11.7
Tetracycline	Doxycycline Hydrochloride	2.5	84.0	5.8
Nitrofurans	Nitofurantoin	15.2	84.6	11.1
Polymyxin	Polymyxin B	0	31.8	0
	Colistin	0	26.9	0
Penicillin	Ampicillin	0	100.0	0

Additional Table S4: Pattern of MDR isolates in different chicken meat samples.

Sample Type	MDR isolates	Non-MDR isolates	Total isolates	Chi-square (P-value)
Good Sanitation	19	8	27	1.92 (0.16)
Poor Sanitation	63	13	76	
Total	82	21	103	

Additional Table S5: Pattern of ESBL producer isolates in different chicken meat samples.

Sample Type	ESBL producer	Non-ESBL producer	Total isolates	Chi-square (P-value)
Good Sanitation	10	17	27	0.04 (0.82)
Poor Sanitation	30	46	76	
Total	40	63	103	

Additional Figure A1: Isolation of *E. coli* from chicken meat (Sample no. B-32)

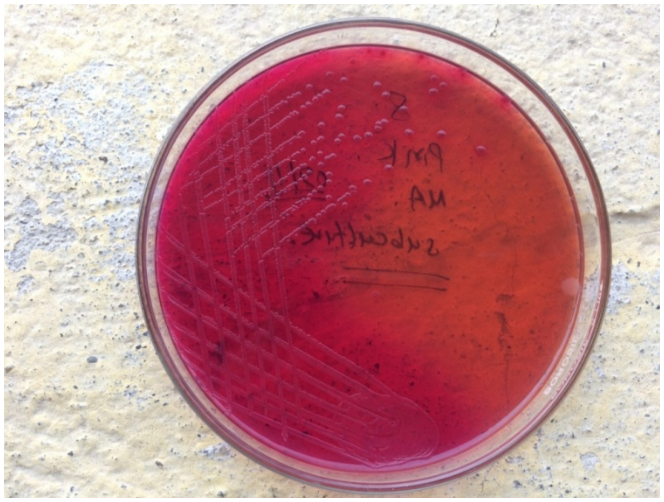

Fig. A1

Additional Figure A2: Antibiotic susceptibility test of E.coli (Sample no. B-32)

(Resistant: Doxycycline Hydrochloride, Ceftazidime; Sensitive: Gentamicin, Ciprofloxacin, Nalidixic acid)

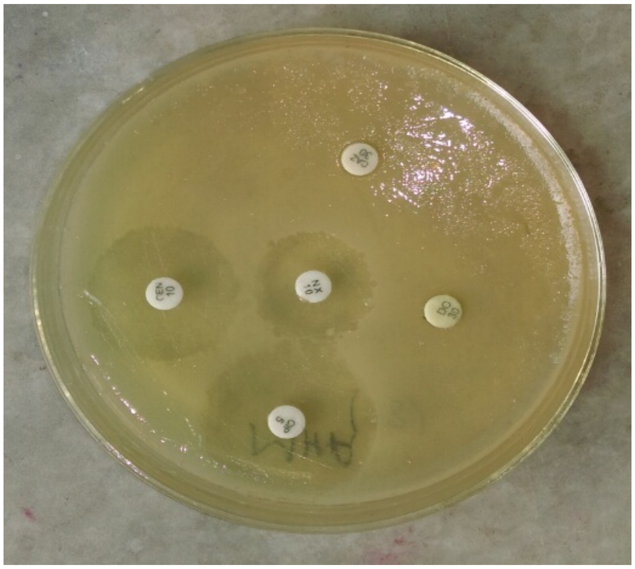

Fig. A2

Additional Figure A3: Detection of ESBL producing *Citrobacter* spp. by Combination disk method (Sample no: B-18)

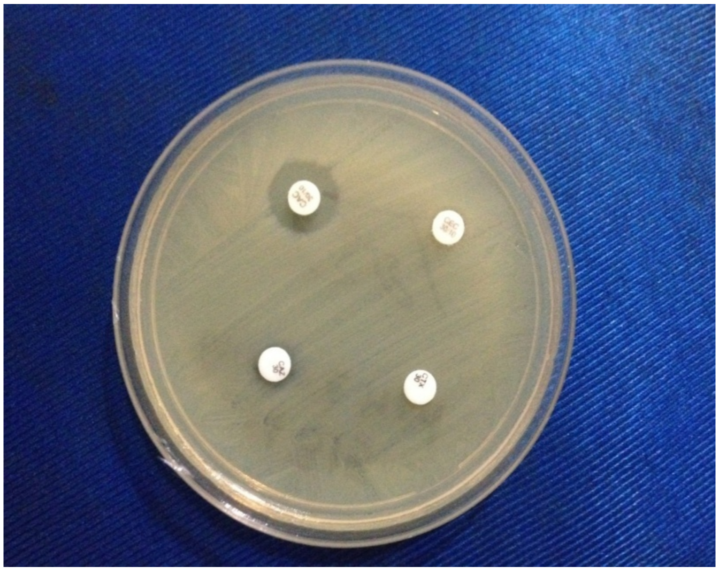

Fig. A3
